# The Phospholipid:Diacylglycerol Acyltransferase Lro1 Is Responsible for Hepatitis C Virus Core-Induced Lipid Droplet Formation in a Yeast Model System

**DOI:** 10.1371/journal.pone.0159324

**Published:** 2016-07-26

**Authors:** Shingo Iwasa, Naoko Sato, Chao-Wen Wang, Yun-Hsin Cheng, Hayato Irokawa, Gi-Wook Hwang, Akira Naganuma, Shusuke Kuge

**Affiliations:** 1 Department of Microbiology, Faculty of Pharmaceutical Sciences, Tohoku Medical and Pharmaceutical University, Sendai, Miyagi, 981–8558, Japan; 2 Laboratory of Molecular and Biochemical Toxicology, Graduate School of Pharmaceutical Sciences, Tohoku University, Sendai, Miyagi, 980–8578, Japan; 3 Institute of Plant and Microbial Biology, Academia Sinica, Nangang, Taipei, 11529, Taiwan; Fundação Oswaldo Cruz, BRAZIL

## Abstract

Chronic infection with the hepatitis C virus frequently induces steatosis, which is a significant risk factor for liver pathogenesis. Steatosis is characterized by the accumulation of lipid droplets in hepatocytes. The structural protein core of the virus induces lipid droplet formation and localizes on the surface of the lipid droplets. However, the precise molecular mechanisms for the core-induced formation of lipid droplets remain elusive. Recently, we showed that the expression of the core protein in yeast as a model system could induce lipid droplet formation. In this study, we probed the cellular factors responsible for the formation of core-induced lipid-droplets in yeast cells. We demonstrated that one of the enzymes responsible for triglyceride synthesis, a phospholipid:diacylglycerol acyltransferase (Lro1), is required for the core-induced lipid droplet formation. While core proteins inhibit Lro1 degradation and alter Lro1 localization, the characteristic localization of Lro1 adjacent to the lipid droplets appeared to be responsible for the core-induced lipid droplet formation. RNA virus genomes have evolved using high mutation rates to maintain their ability to replicate. Our observations suggest a functional relationship between the core protein with hepatocytes and yeast cells. The possible interactions between core proteins and the endoplasmic reticulum membrane affect the mobilization of specific proteins.

## Introduction

Nearly 3% of the world’s population is chronically infected with the hepatitis C virus (HCV) [1], which is a major risk factor for liver cirrhosis and hepatocellular carcinoma [2]. Chronic infection with HCV causes the abnormal accumulation of significant levels of liver lipids (steatosis). Steatosis has been significantly associated with hepatocellular carcinomas in HCV-infected patients [3]. Steatosis is characterized by the accumulation of liver lipid droplets (LDs), which are essential for RNA replication and HCV particle formation [4, 5].

The positive-stranded RNA genome of HCV encodes an approximately 3,000 amino acid polyprotein. Following translation, the amino-terminal region of the polyprotein is cleaved by a host signal peptidase to yield three structural proteins (core, E1 and E2). The other regions of the polyprotein are processed by viral proteases to yield seven nonstructural proteins [6]. The C-terminal region of the core protein is further processed by a host signal peptidase to generate a mature core (amino acid 1 to 177, hereafter called core), which then enables the translocation to LDs. The core consists of two domains (D1 and D2). The D1 domain is responsible for RNA binding, whereas the D2 domain anchors to the surfaces of the ER and LDs [7, 8]. The core is believed to have an important role in pathogenesis, as shown by the formation of steatosis and hepatocellular carcinomas in core-transgenic mice [9]. Furthermore, interactions between the core and various factors involved in lipogenesis have been reported [10, 11]. A triglyceride (TAG) synthetic enzyme, acyl-CoA:diacylglycerol O-acyltransferase (DGAT), is required for the core to translocate from the ER to LDs. However, the activity of DGAT1 is not affected by the core [10]. The presence of the core on the surface of LDs impairs LD turnover by inhibiting the activity of adipose triglyceride lipase (ATGL) [12]. It has been suggested that the core enhances the disequilibrium of ATGL and its cofactor and that the core can alter the intracellular vesicular trafficking of these factors [11]. However, the mechanism underlying the effect of the core on these factors, such as the interactions between the core and the endoplasmic reticulum (ER) membrane that lead to the altered translocation of these factors, remains obscure.

Yeast (*Saccharomyces cerevisiae*) is a viable model system to study neutral lipid homeostasis for higher eukaryotes [13, 14]. Recently, we showed that the expression of the core protein in yeast reproduced several characteristics of the core protein that are observed in mammalian cells [15]. The core protein is localized to the cytoplasmic side of the ER and enhances LD formation in yeast cells. Our observations suggested a functional analogy of the core between hepatocytes and yeast cells, namely in the intrinsic characteristics of the core.

LDs form when sterol ester (SE) and/or TAG accumulate and are surrounded by phospholipid monolayers. The syntheses of cellular SE and TAG are catalyzed by the acyltransferase family of proteins. TAG is synthesized by DGAT in mammalian cells. In yeast, TAG is synthesized by a DGAT homologue, encoded by the gene *DGA1*, and a phospholipid:diacylglycerol O-acyltransferase (PDAT), encoded by the gene *LRO1* [16]. Additionally, two acyl-CoA:cholesterol O-acyltransferase (ACAT)-related enzymes (Are1 and Are2) are responsible for SE synthesis [17]. Cells with these enzymes (Dga1, Lro1, Are1 and Are2) disrupted failed to form LDs, whereas the presence of at least one of these enzymes permits LD formation [18].

In this work, we attempted to elucidate the genes responsible for the effect of the core protein on lipid droplet formation in yeast cells. We demonstrated that Lro1 was required for core-induced LD formation. The stability of Lro1 was extended, and the localization of Lro1 was altered in response to core expression. We further discussed a possible mechanism underlying the core-induced ER changes that may lead to LD expansion.

## Materials and Methods

### Yeast media, culture and strain construction

Yeast cells were grown at 30°C in synthetic raffinose medium (yeast nitrogen base without amino acids, BD Difco, NY, USA) supplemented with 2% raffinose (SRM) and with the following amino acid and nucleotide supplements [19]: 40 μg/ml adenine hemisulfate, 20 μg/ml L-arginine monohydrochloride, 100 μg/ml L-aspartic acid, 100 μg/ml L-glutamic acid monosodium salt, 68 μg/ml L-lysine monohydrochloride, 20 μg/ml L-methionine, 50 μg/ml L-phenylalanine, 375 μg/ml L-serine, 200 μg/ml L-threonine, 30 μg/ml L-tyrosine, 150 μg/ml L-valine, 20 μg/ml L-histidine, 60 μg/ml L-leucine and 20 μg/ml uracil. L-tryptophan was omitted. To induce the *GAL1* promoter-dependent transcription, we added galactose at a final concentration of 3% to exponentially growing yeast cultures and incubated the cultures for 3 h [20]. The construction of the yeast strains was carried out as previously described [21]. BY4742 (derived from S288C; EUROSCARF) was used as the wild-type control cells. All the mutants were isogenic for BY4742; a list of these strains is provided in S1 Table. Single and double disruptions were created by transfecting each DNA fragment containing the *are1*Δ::*LEU2*, *are2*Δ::*HIS3*, *dga1*Δ::*KAN* or *lro1*Δ::*HYG* loci, which were isolated from CWY3768 [14] cells by PCR. The genomic replacement of *LRO1* and *HMG2* with those fused with mCherry or GFP were constructed using a PCR-based method as previously described [22].

### Construction of plasmids

A multi-copy plasmid (2 μm Ori, *URA3* marker) with the *GAL1* promoter (pKT10-GAL) was used to express the core proteins. A cDNA region of the core (1–177 aa) was isolated by PCR from HCV genotype 1b (GenBank: AY045702) [23] and cloned under the *GAL1* promoter of pKT10-GAL (pKT10-GAL-core) [20]. To construct pRS315, a CEN-based low copy plasmid with a *LEU2* marker, [24] carrying DGA1-mCherry and LRO1-Myc, we isolated the genomic fragments of the fusion gene loci from CWY5135 and CWY3773 [14] by PCR and cloned them into pRS315. The core D2 region (the D2 domain and the residual C-terminus, aa 118–177) was fused to the C-terminus of DsRed-Monomer (Clontech, Takara Bio Company, Kyoto, Japan) and GFP and cloned as above to generate pKT10-GAL-DsRed-core and pKT10-GAL-GFP-core, respectively.

### Preparation of cell lysates and Western blotting

The preparation of yeast lysates and Western blotting were performed as previously described [20]. We used primary antibodies specific for actin (sc-1616; Santa Cruz Biotechnology, Santa Cruz, CA, USA), Myc (M192-3S; MBL, Nagoya, Japan), and the core (515S) [25]. The core was characterized using the Chemi-Lumi One L (Nakalai Tesque, Kyoto, Japan) or Immobilon^TM^ Western detection reagent (EMD Millipore, Billerica, MA, USA) and a VersaDoc^TM^ chemiluminescence detector (Bio-Rad Laboratories, Inc., Hercules, CA, USA; dynamic range: 10^5^) and was quantified using the Quantity One^TM^ software (Bio-Rad).

### The detection and quantitation of LDs and fluorescence microscopy

Live yeast cells were stained with 4,4-difluoro-1,3,5,7,8-pentamethyl-4-bora-3a,4a-diaza-*s*-indacene (BODIPY 493/503, Invitrogen, ThermoFisher Scientific, Waltham, MA, USA) as previously described [14]. The fluorescent images of the LDs and the fluorescent protein fusions of the core, Hmg2, Lro1 and Dga1 were captured using a fluorescence microscope, as described previously [14]. The number of LDs was counted in the images produced by the maximal projection of ten Z-optical sections (spaced at a thickness of 0.56 μm). We used the same capture condition for the fluorescent images for each of the experiments. There were no differences in contrast enhancement (BODIPY fluorescence) between various strains in each of the experiments.

### Lipid analysis

Phospholipid extraction was performed using the Folch procedure [26]. Briefly, 20 OD_600_ units of cells were collected and treated with 10 mM NaN_3_ for 10 min. The cells were pelleted and resuspended in approximately 20 μl of the original medium. The cells were lysed by vortex for 10 min in 330 μl of methanol with 100 μl of glass beads, and then, 660 μl of chloroform was added. The supernatants from the centrifugation were supplemented with 200 μl of 0.9% sodium chloride. After another centrifugation, the lower organic phase was dried using a Speedvac and then dissolved in chloroform:methanol (2:1). The lipids were analyzed by thin layer chromatography as previously described [27]. To quantify TAG, we further performed liquid chromatography-mass spectrometry (LC-MS) analysis using an Orbitrap mass spectrometer (Orbitrap Elite, Thermo Fisher Scientific, Bremen, Germany) coupled with an UHPLC system (ACQUITY UPLC, Waters, Milford, MA). Solvent A was 10 mM ammonium acetate, pH 5.0 and 40% acetonitrile as the aqueous phase, and solvent B is 10 mM ammonium acetate, pH 5.0 and 10% acetonitrile in isopropyl alcohol as the mobile phase. The lipids were separated with a CSH C18 column (1.8 μm, 2.1 mm x 100 mm, Waters, Milford, MA) at a flow rate of 450 μl/min using a gradient of 40–99.5% solvent B over 0–10 minute. For TAG profiling, the mass spectrometer was operated in positive ion mode, and the full FT-MS scan data (*m*/*z* 100–1200, Res = 30,000) were analyzed and quantified with the Xcalibur software (Thermo Scientific, Waltham, MA). Linoleic acid-d4 (Cayman Chemical, MI) was added to the lipid extraction step to serve as an internal control for quantification.

### Statistical analysis

All experiments were repeated at least three times, and multiple independent replicates (more than n = 3, indicated in the figure legends) were performed for each experiment. The data are presented as the mean of the replicates with the standard error of the mean (SEM).

## Results

### Lro1 is responsible for core-induced LD formation

**Fig 1:**
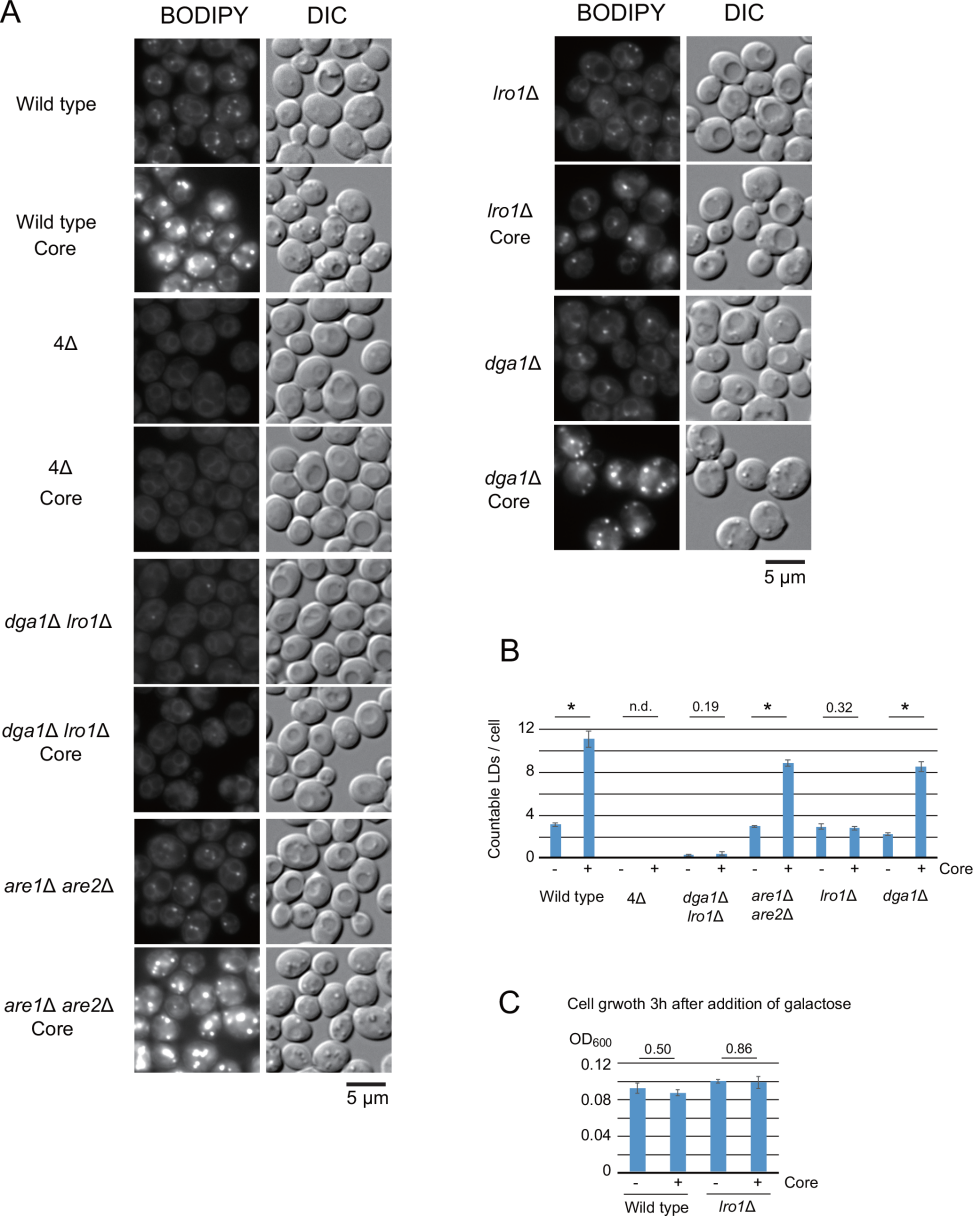
The core-induced LD level is mediated by the genes responsible for TAG synthesis. (A) LD levels in yeast mutants with defects in neutral lipid synthesis. The genotypes of the yeast strains are indicated in the figure. The wild type (BY4742) and its isogenic mutants, dga1Δ lro1Δ (BY4742 *dga1*Δ::*KAN lro1*Δ::*HYG)*, are1Δ are2Δ (BY4742 *are1*Δ::*LEU2 are2*Δ::*HIS3)*, dga1Δ (BY4742 *dga1*Δ::*KAN*) and lro1Δ (BY4742 *lro1*Δ::*HYG*). Δ4 indicates a quadruple disruption mutant (CWY3768: BY4742 *are1*Δ::*LEU2 are2*Δ::*HIS3 dga1*Δ::*KAN lro1*Δ::*HYG*), which lacks all four neutral lipid synthesizing genes. Cells carrying the empty vector pKT10-GAL (upper panels) or the pKT10-GAL-core plasmid (lower panels, designated as “Core”) were cultured in raffinose/galactose-containing medium (SRM + Gal) for 3 h. The LDs in live yeast cells were stained with BODIPY 493/503 and analyzed by fluorescent microscopy. The differential interference contrast images (DIC) and BODIPY 493/503 fluorescent images (BODIPY) are shown. Scale bars: 5 μm. The expression level of core in each of disruption mutant is not significantly different (S1 Fig). (B) The number of LDs/cell was counted in images produced by the maximal projection of ten z-sections in 5 μm thickness (S2 Fig). The data are represented as the mean ± SEM (N = 5, five countings of LD number in 10 cells: total 50 cells). “*” indicates significance (*P* < 0.01), and other *P* values are indicated. No LDs were detected in Δ4 cells (n.d.). (C) The growth rate of wild-type cells and *lro1*Δ cells carrying the empty vector pKT10-GAL (-) or the pKT10-GAL-core (+) upon the induction of core expression. Galactose (3%) was added to exponentially growing yeast cell cultures in SRM (OD_600_ = 0.1). The cultures were further cultured for 3 h. The growth levels after the addition of galactose are shown. The *P* values are indicated.

We determined the LD levels in yeast cells using BODIPY 493/503, a lipophilic dye that specifically stains neutral lipids. As we recently reported [15], the induction of the expression of the core protein for 3 h significantly enhanced LD levels in the fluorescent images (Fig 1A, Wild type, Core). As shown in Fig 1B, the countable number of LDs per cell was significantly increased by the expression of the core (Wild type, +). To identify the gene responsible for the core-dependent increase in LD formation, we examined several mutants that lack the genes for neutral lipid syntheses. *ARE1* and *ARE2* are responsible for SE synthesis, and *DGA1* and *LRO1* are responsible for TAG synthesis. The expression levels of the core in each of the disrupted mutant cells were similar (S1 Fig). As shown in Fig 1A and 1B, the yeast strain lacking all four genes (a quadruple mutant, 4Δ), which was previously shown to be defective in forming LDs [18], also failed to form LDs by the core expression. Interestingly, the simultaneous loss of Dga1 and Lro1, but not Are1 and Are2, impaired the core-dependent induction of LD formation. We observed an increase in the punctate fluorescence of LDs by the core expression in wild-type and *are1*Δ *are2*Δ cells, but not in the quadruple mutant (4Δ) or *dga1*Δ *lro1*Δ cells (Fig 1A and 1B). Furthermore, the *LRO1* single knockout had a markedly reduced level of the core-induced LD formation, whereas no changes were observed in the *DGA1* single knockout (Fig 1A and 1B).

It should be noted that morphological alterations in the cell surrounding LDs were observed in wild-type cells, *are1*Δ *are2*Δ cells and *dga1*Δ cells when the core was induced (Fig 1A, DIC images), but not in 4Δ cells, *dga1*Δ *lro1*Δ cells and *lro1*Δ cells. Thus, this morphological alteration is likely to be Lro1-dependent.

Neutral-lipid homeostasis is affected by the balance between lipid supply and consumption, and thus, carbon source availability and cell growth [13]. We examined the effect of core expression on yeast growth in the culture condition used above. As shown in Fig 1C, the growth of wild-type cells and *lro1*Δ cells were not affected by the 3 h induction of core expression. A prolonged induction of the core expression (6 h) showed a mild growth retardation for wild-type cells and *lro1*Δ cells (S2B Fig). These results revealed that the Lro1-dependent enhancement of LD levels by core expression was independent of cell growth.

**Fig 2:**
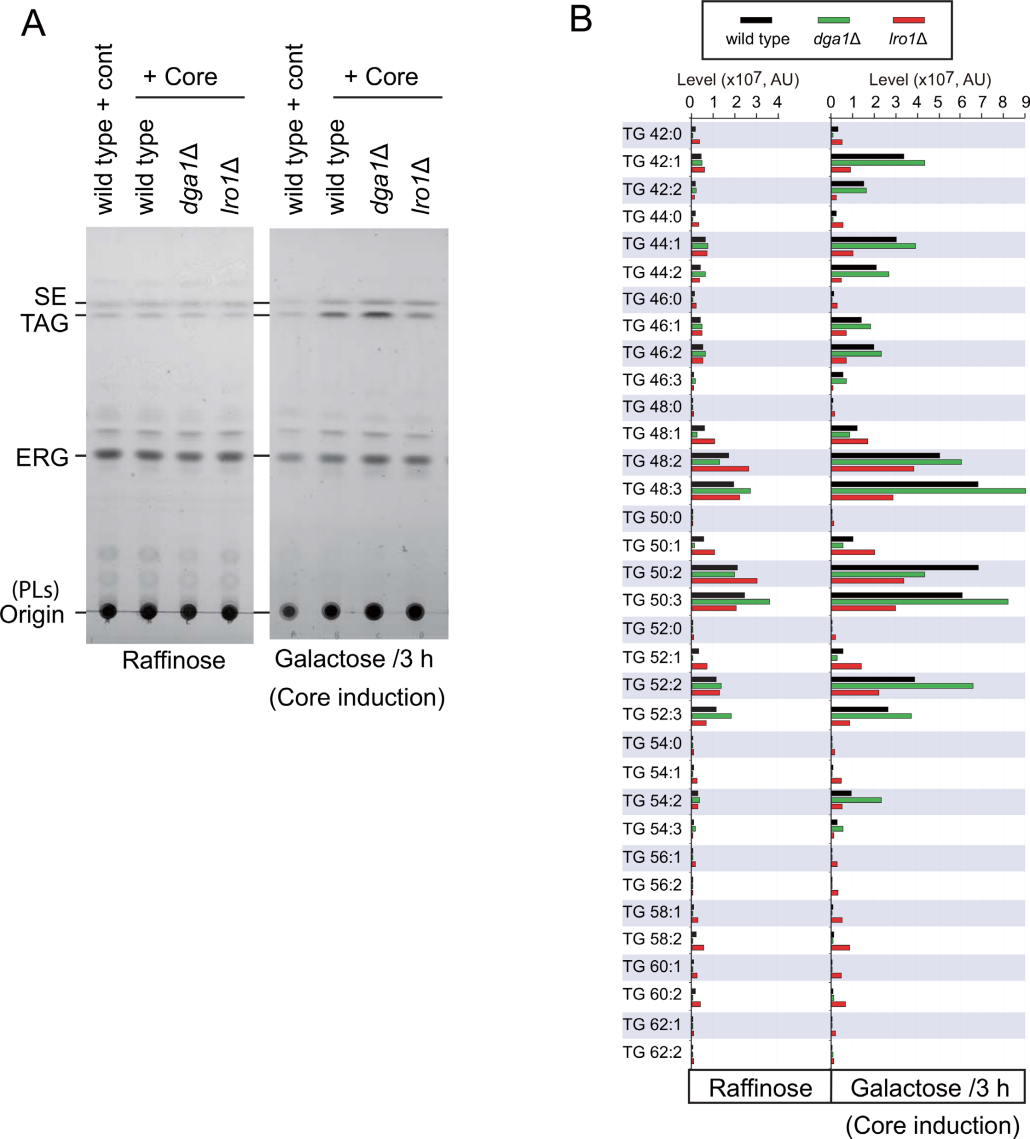
Lipid analysis for the core-expressing yeast cells. (A) TLC analyses of the neutral lipids were performed as described previously [27]. The positions of ergosterol (ERG), sterol ester (SE) and triacylglycerol (TAG) are indicated. Lipids species found in wild-type cells, dga1Δ cells and lro1Δ cells carrying the pKT10-GAL-core plasmid before (raffinose) and after 3 h of culture with galactose (Galactose) compared by TLC analysis. (B) TAG species found in wild-type (black bars), dga1Δ (green bars) and lro1Δ (red bars) cells carrying the pKT10-GAL-core plasmid before (raffinose) and after 3 h of culture with galactose (Galactose) by LC/MS analysis are depicted, and their abundances are compared. The signal intensity unit for the mass spectrometry detector is indicated as arbitrary unit (AU) for quantification of the abundance of specific m/z of the indicated lipid species.

As shown in Fig 2A, lipid analyses demonstrated that the TAG level was enhanced in wild-type and *dga1*Δ cells by core expression, whereas the levels of phospholipids (PLs), ergosterol (ERG) and SE were unaffected. Lipid profiles using the liquid chromatography also showed that the levels of TAG species, but not the levels of lysophospholipids, PLs and diacylglycerol (DAG), were enhanced in wild-type and *dga1*Δ cells by core expression (S3 Fig). Most of the TAG species that were upregulated by the core expression were mainly Lro1-dependent (Fig 2B). These results suggested that Lro1 was the enzyme most responsible for the core-dependent induction of the upregulation of TAG levels, resulting in the induction of LDs.

### Core suppresses ER-associated degradation (ERAD) of Lro1 but increases LDs in an ERAD-independent manner

**Fig 3:**
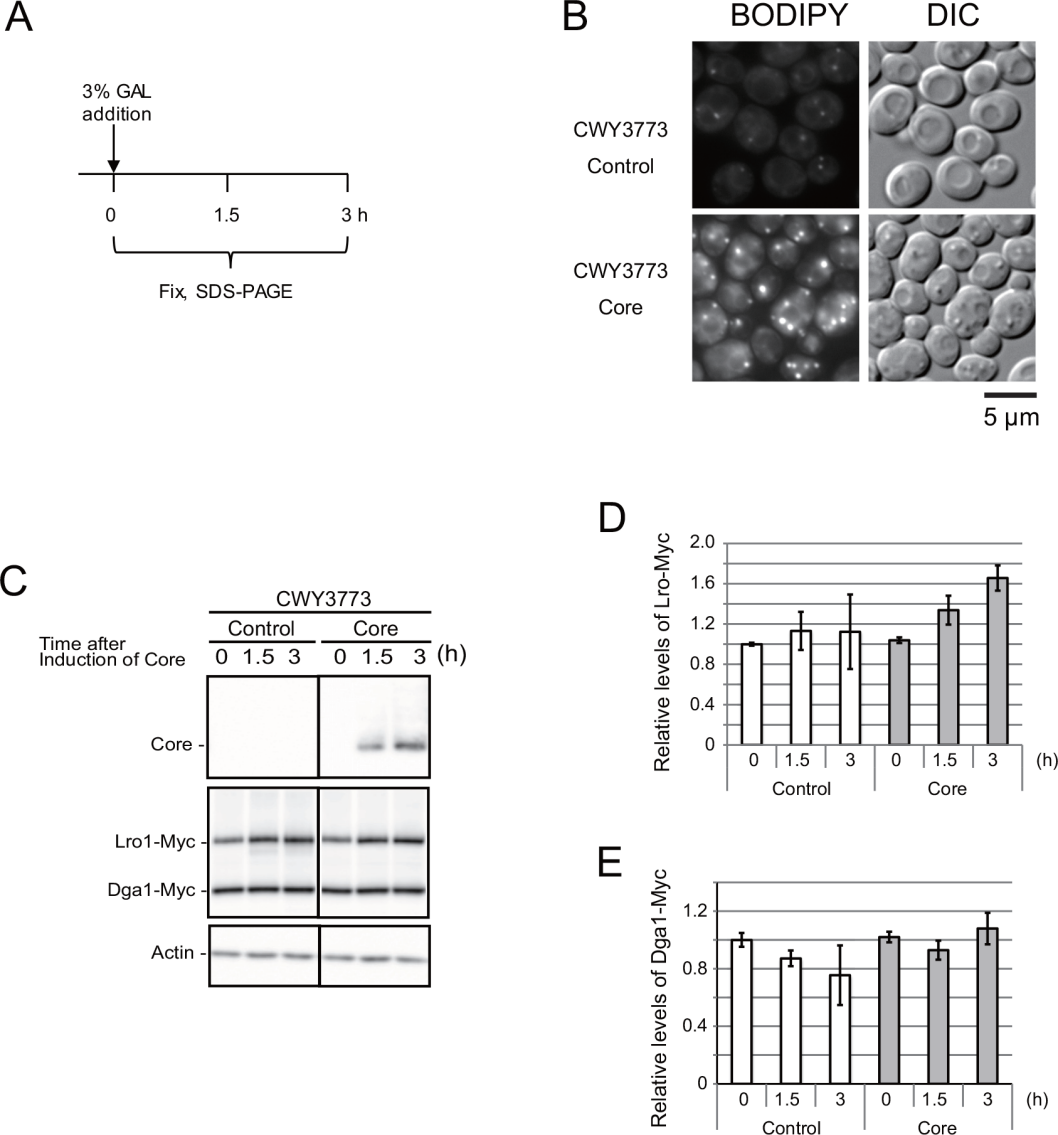
Lro1 level is enhanced by core. (A) The scheme for sample preparation. Galactose was added at time 0 to induce core expression. The cells were collected and fixed at the indicated time (0, 1.5 and 3 h). (B) LDs were successfully induced in CWY3773 (BY4742 *LRO1-13xMyc*::*LEU2 DGA1-13xMyc*::*HIS3*) cells expressing both Lro1-Myc and Dga1-Myc with core expression. CWY3773 cells were transformed with the empty vector pKT10-GAL (Control) or the pKT10-GAL-core plasmid (Core), and cultured for 3 h with galactose. (C) The Western blot of Lro1-Myc and Dga1-Myc. See S5 Fig for the original data. (D) and (E) The intensity was normalized using the actin intensity. The relative levels of Lro1-Myc (D) and Dga1-Myc (E) are indicated (N = 3).

To examine whether core expression affected the Lro1 protein level, we monitored the time-dependent changes in the protein levels of Lro1 and Dga1 after the induction of core expression (Fig 3A) in yeast cells (CWY3773) that expressed both Myc-tagged Lro1 (Lro1-Myc) and Myc-tagged Dga1 (Dga1-Myc) under their own promoters of the respective gene loci [14]. We confirmed that the expression of the core in CWY3773 conferred the induction of LD formation (Fig 3B). Furthermore, the quadruple mutant that expressed only Lro1-myc successfully enhanced LD formation in response to core expression (S4 Fig), suggesting that the 13xMyc tag at the C-terminal end of Lro1 exhibited a core-dependent phenotype. Morphological alterations were also observed in these Lro1-Myc expressing cells (Fig 3B and S4 Fig). Using Lro1-myc, we monitored the effect of the core expression on the Lro1 level. The Lro1-Myc level slightly increased in response to core expression (Fig 3C and 3D). In contrast, the Dga1-Myc level was not affected by the core expression (Fig 3C and 3E).

**Fig 4:**
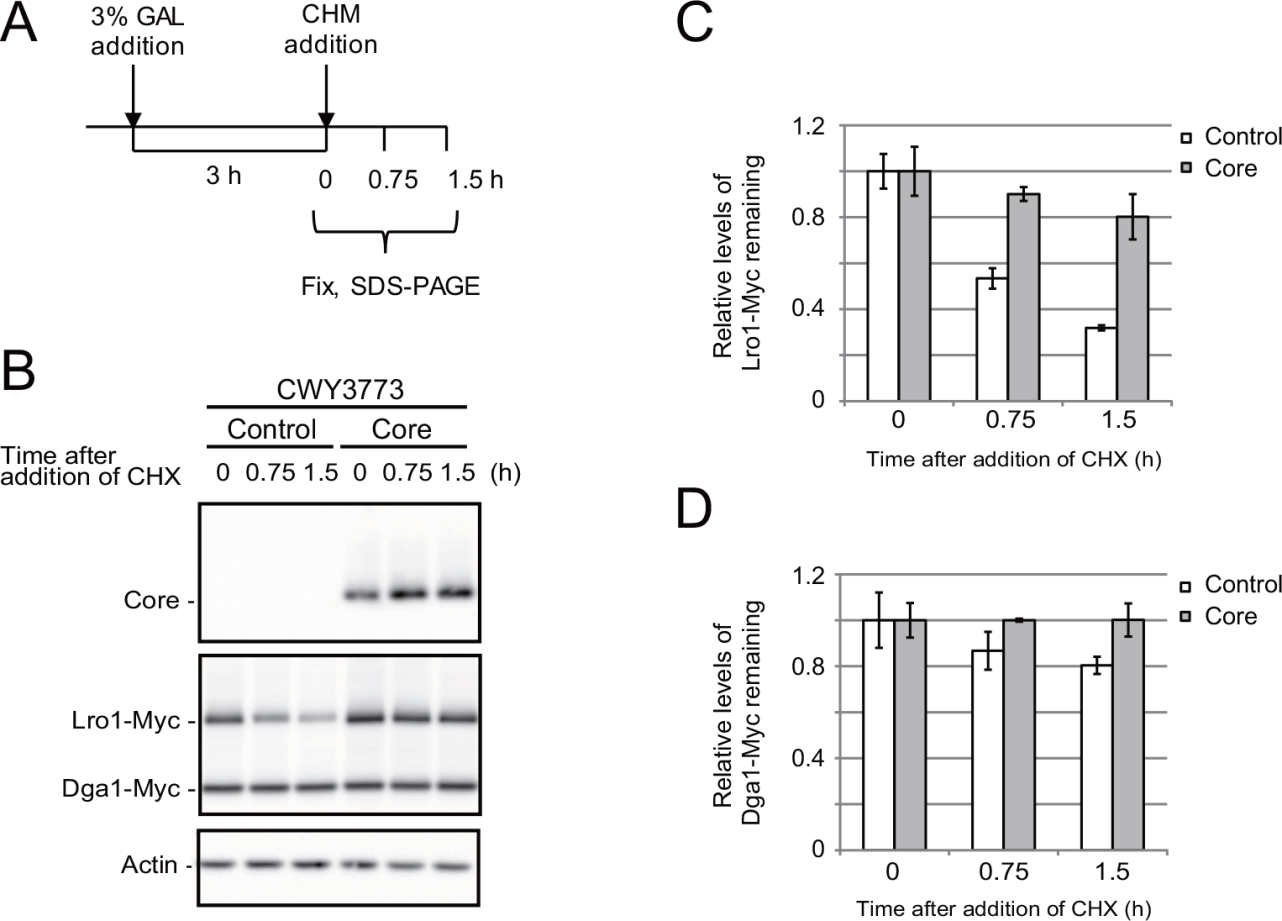
Degradation of Lro1 is inhibited by core. (A) The scheme for sample preparation. The core expression was induced by the addition of galactose for 3 h. Then, protein synthesis was stopped by adding 0.3 mM cycloheximide (CHX) at the time 0. The cells were fixed at the indicated time (0, 0.75 and 1.5 h). (B) The CHX chase Western blot of Lro1-Myc and Dga1-Myc. CWY3773 (BY4742 *LRO1-13xMyc*::*LEU2 DGA1-13xMyc*::*HIS3*) cells expressing both Lro1-Myc and Dga1-Myc were transformed with the empty vector pKT10-GAL (Control) or the pKT10-GAL-core plasmid (Core). See S5 Fig for the original data. (C) and (D) The intensity was normalized using the actin intensity. The relative levels of intensity of Lro1-Myc (C) and Dga1-Myc (D) are shown (N = 3).

Next, we determined the degradation rates of the proteins Lro1-Myc and Dga1-Myc in core-expressing cells after protein synthesis had been inhibited by cycloheximide (Fig 4A). As shown in Fig 4B–4D, the cycloheximide-chase experiment suggested that the degradation rate of Lro1-Myc was significantly higher than that of Dga1-Myc in cells without core expression. However, the expression of the core protein inhibited the degradation of Lro1-Myc. Previous reports have indicated that Dga1 is a short-lived protein, whereas Lro1 is relatively stable during the late exponential growth phase in glucose medium [14]. This discrepancy suggests that the fates of Dga1 and Lro1 may be dependent on different carbon sources and culture conditions.

**Fig 5:**
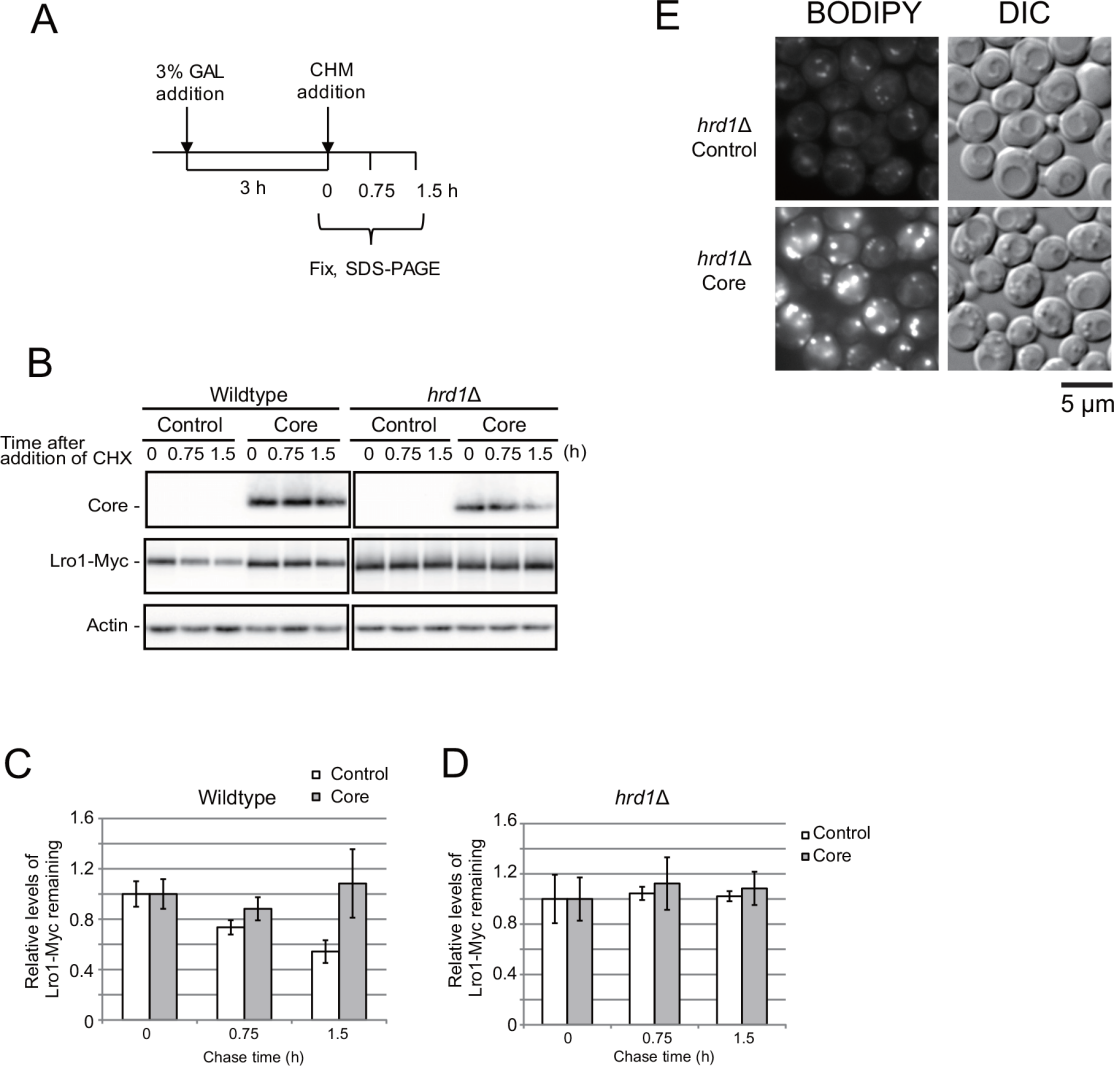
Core-induced LD formation is not mediated by inhibition of Hrd1-dependent Lro1 degradation. (A) The scheme for sample preparation is the same as Fig 4A. (B) The CHX chase western blotting of Lro1-Myc and Dga1-Myc in wild-type and hrd1Δ cells. BY4742 and BY4742 *hrd1*Δ cells were transformed with the pRS315-Lro1-Myc plasmid and the empty vector pKT10-GAL (Control) or pKT10-GAL-core (Core). pRS315 is a CEN-based plasmid containing *LEU2* as a selection marker [24], and these vectors each contained their own promoter for *LRO1* and *DGA1*. See S5 Fig for the original data. (C) and (D) The intensity was normalized using the actin intensity. The relative levels of the intensity of wild-type cells (C) and hrd1Δ cells (D) are shown (N = 3). (E) The LD levels in hrd1Δ cells were determined as described in the Fig 1 legend.

By extending the Lro1 half-life, which was induced by the core protein, we may increase LD formation. Hrd1 ubiquitin ligase is required for the degradation of proteins embedded in the ER membrane [28]. The loss of Hrd1 (*hrd1*Δ cells) impaired the rapid degradation of Lro1 (Fig 5A–5D, S5 Fig) and suggested that the turnover of Lro1-Myc was mediated by the Hrd1-dependent ERAD pathway. Nevertheless, the disruption of *HRD1* did not affect the core-dependent increase in the LD level (Fig 5E), despite the presence of constitutive high Lro1-Myc levels in the *hrd1*Δ cells (Fig 5B).

### The Lro1 distribution changed in response to core expression

**Fig 6:**
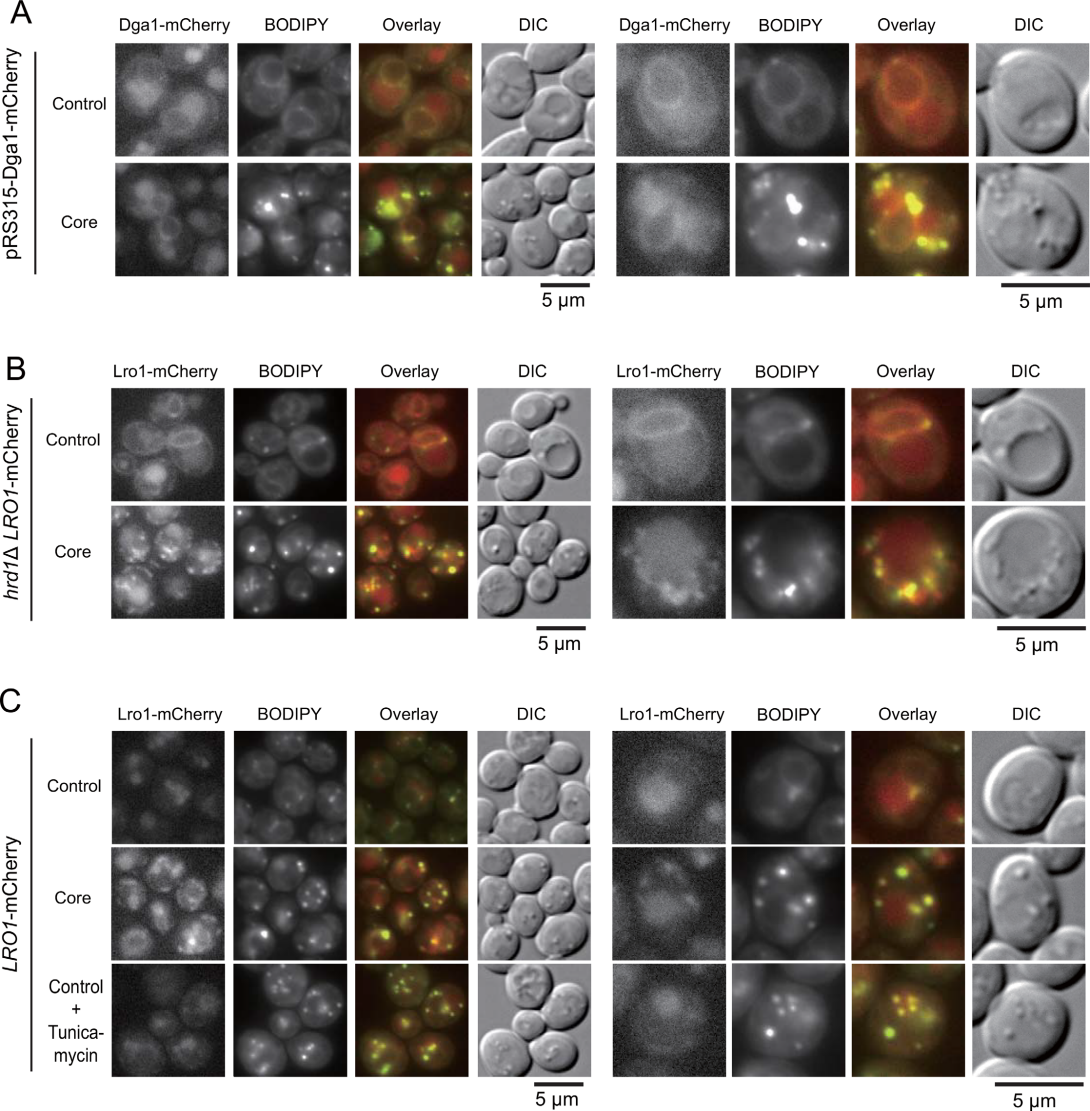
Changes in the distribution of Lro1-mCherry, but not Dga1-mCherry, in response to the core. (A) The distribution of Dga1 in cells expressing the core. Wild-type cells carrying pRS315-Dga1-mCherry with the empty vector pKT10-GAL (Control) or the pKT10-GAL-core plasmid (Core) were stained with BODIPY 493/503 after the cells were cultured in SRM + Gal medium for 3 h. The fluorescent images of BODIPY 493/503-stained LDs and Dga1-mCherry were examined using fluorescent microscopy. Images of mCherry, BODIPY 493/503, and the overlay of mCherry (red) and BODIPY (green), and DIC are shown. Representative images were magnified (right panels). Scale bars: 5 μm. (B) Changes in the distribution of Lro1 by the core expression. The same experiments in (A) were performed with hrd1Δ cells expressing Lro1-mCherry using an endogenous promoter of the chromosomal locus (hrd1Δ LRO1-mCherry: BY4742 *hrd1*Δ::*KAN LRO1-mCherry*::*LEU2*). (C) The expression of the core, but not of tunicamycin, altered the distribution of Lro1-mCherry in wild-type cells expressing Lro1-mCherry using an endogenous promoter of the chromosomal locus (LRO1-mCherry: BY4742 *LRO1-mCherry*::*LEU2*). The cells carrying the empty vector were treated with tunicamycin (4 μg/ml) in SRM + Gal medium for 3 h (the lowest panels). Scale bars: 5 μm.

Because the quantitative enhancement of Lro1 by the core protein did not seem to be directly responsible for the core-dependent increase in LD levels, we speculated that the distribution of Lro1 might be affected by core expression. We examined the effect of core expression on the distribution of Dga1-mCherry and Lro1-mCherry, both of which are functional in terms of TAG synthesis [14]. The distribution of Dga1-mCherry (Fig 6A) in yeast cells growing in raffinose/galactose was perinuclear and cortical shaped and also in vacuoles, and the distribution was unaltered by core expression (Fig 6A). Interestingly, as shown in Fig 6B and 6C, mCherry-labeled Lro1 was also perinuclear and cortical shaped, especially *hrd1*Δ cells, in which the fluorescent level of Lro1-mCherry was enhanced (Fig 6B). The Lro1-mCherry fluorescence was shown as punctuated and laminar structures in response to core expression in the proximity of the LDs. Although a portion of Lro1-mCherry also localized to the vacuole, the vacuole distribution of Lro1-mCherry was unaltered in response to the core expression (Fig 6B and 6C).

To examine whether the presence of LDs triggered the redistribution of Lro1-mCherry, even in the absence of the core protein, we observed Lro1-mCherry in cells in which LDs were induced by tunicamycin treatment. As an ER-stress inducer, tunicamycin can increase LD formation in yeast [29] and in human hepatoma cells [30]. As shown in Fig 6C, tunicamycin treatment in the absence of the core protein successfully induced LD formation but had no apparent effect on the Lro1-mCherry distribution. These results suggested that the relocalization of Lro1-mCherry did not seem to be determined by the formation of LDs.

### The core D2 region is responsible for LD formation and its localization close to LDs and Lro1

**Fig 7:**
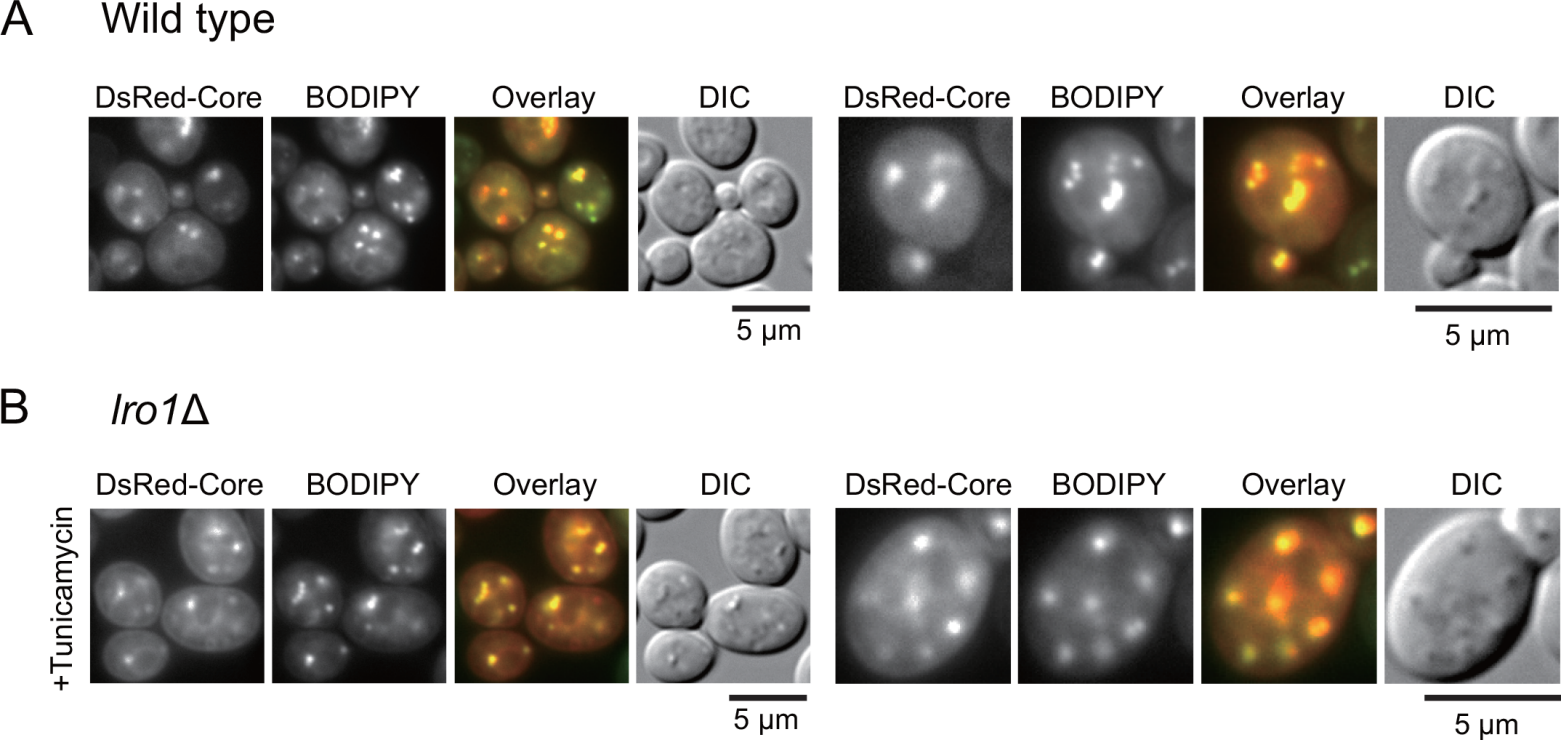
Localization of DsRed-core and LDs in wild-type and tunicamycin-treated *lro1Δ* cells. (A) Wild-type cells carrying the pKT10-GAL-DsRed-core plasmid were cultured in SRM + Gal medium for 3 h. Cells were stained with BODIPY 493/503. The images of DsRed, BODIPY 493/503, the overlay of DsRed (red) and BODIPY (green), and DIC are shown. The representative images were magnified (right panels). (B) Lro1Δ (BY4742 *lro1*Δ::*HYG*) cells carrying the pKT10-GAL-DsRed-core plasmid were cultured in SRM + Gal medium and tunicamycin (4 μg/ml) for 3 h and were stained with BODIPY 493/503. Fluorescent images were taken as described above. Scale bars: 5 μm.

As in mammalian cells [7, 8], the D2 region, (the D2 domain and the residual C-terminus), has the ability to localize on the periphery of the nucleus [20]. We examined whether the core D2 region could co-localize with LDs, as observed in mammalian cells [8]. As shown in Fig 7A, DsRed-core (core amino acids 118–177 fused with DsRed) accumulated as punctate structures and localized close to the BODIPY 493/503-stained LDs. Next, we investigated whether the core protein punctate structures adjacent to the LDs were Lro1-dependent. To examine this issue, we induced LD formations using a tunicamycin treatment with the simultaneous induction of DsRed-core in *lro1*Δ cells. As shown in Fig 7B, the DsRed-core colocalized with LDs, which was induced by tunicamycin in *lro1*Δ cells. Thus, we concluded that Lro1 might be unnecessary for the punctate localization of DsRed-core on the adjacent surface of LDs.

**Fig 8:**
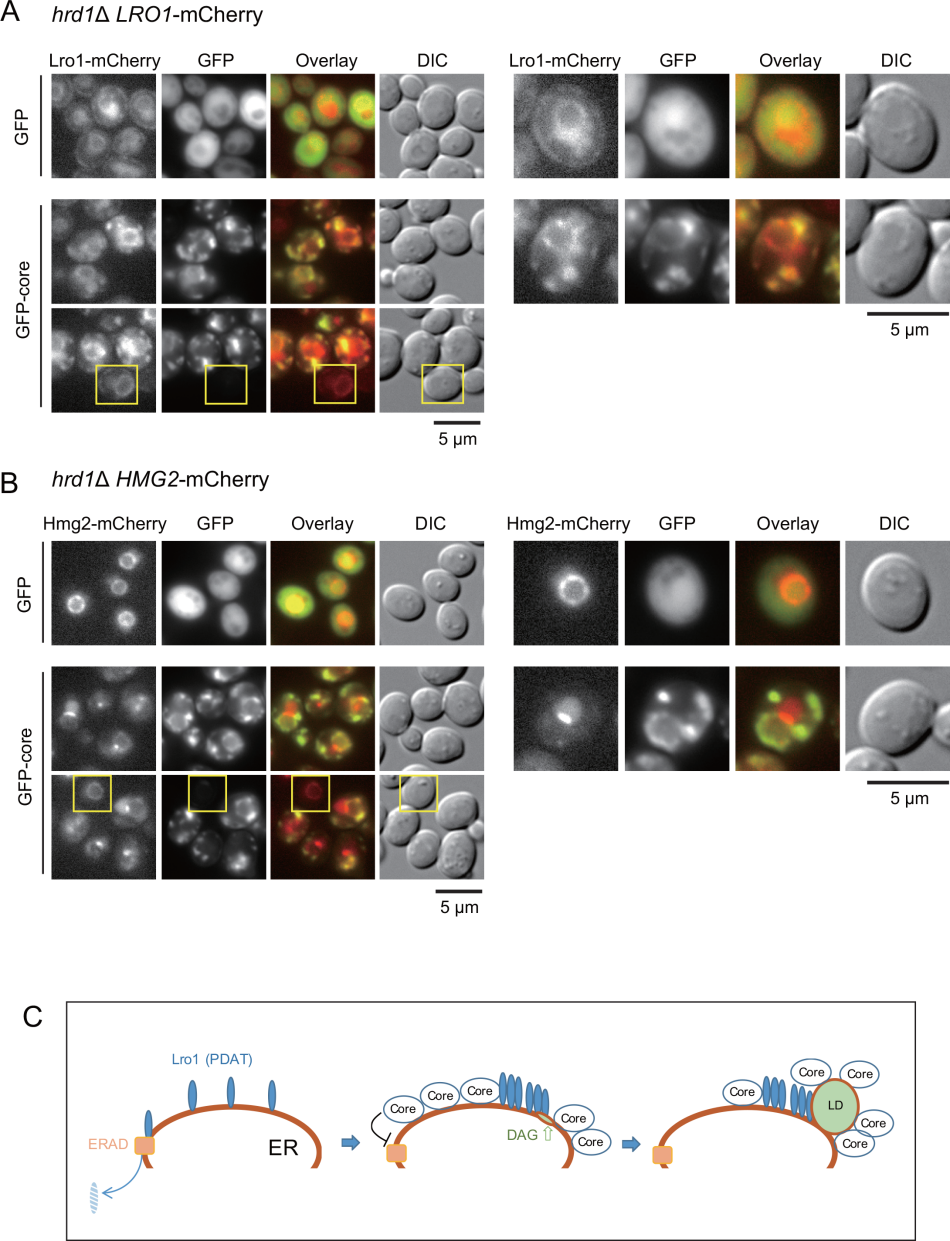
Effect of GFP-core on localization of Lro1-mCherry and Hmg2-mCherry. (A) The colocalization of GFP-core and Lro1-mCherry was observed. Hrd1Δ LRO1-mCherry (BY4742 *hrd1*Δ::*KAN LRO1-mCherry*::*LEU2*) cells carrying the pKT10-GAL-GFP (Control) plasmid or the pKT10-GAL-GFP-core (GFP-core) plasmid were cultured in SRM + Gal medium for 3 h. Fluorescent images of Lro1-mCherry, GFP, the overlay of GFP (green) and mCherry (Red), and DIC are shown. Representative images were magnified (right panels). As an internal control, the yellow box indicates one cell with a distribution of Lro1-mCherry that was not affected in the absence of GFP-core expression. (B) The expression of GFP-core induced the redistribution of Hmg2-mCherry. Hrd1Δ HMG2-mCherry (BY4742 *hrd1*Δ::*KAN HMG2-mCherry*::*LEU2*) cells carrying the pKT10-GAL-GFP plasmid or the pKT10-GAL-GFP-core plasmid were cultured in SRM + GAL medium at 30°C. Because Hmg2 is also a substrate for the Hrd1-dependent ERAD pathway [28], we used the *hrd1*Δ genotype for this experiment. Fluorescent images of GFP and Hmg2-mCherry were taken. Scale bars: 5 μm. (C) A model for core-induced LD formation. The expression of core accumulated at a specific locus on the ER, which inhibits ERAD and triggers Lro1 accumulation. The accumulation of Lro1 induced LD formation.

The above results suggested that both the core protein and Lro1 might have adjacently co-localized on the surface of LDs. We found that the GFP-core was partially colocalized with Lro1-mCherry. Fig 8A shows that Lro1-mCherry was localized in larger patches and the GFP-core surrounded Lro1 patches in punctate structures.

Our previous results suggested that the core protein may affect the ER membrane [20]. To examine whether the punctate localization of core protein affected the ER structure, we examined the localization of the GFP-core in cells expressing the ER membrane protein Hmg2-mCherry. As shown in Fig 8B, the GFP-core expression also altered the localization of Hmg2-mCherry. The Hmg2-mCherry fluorescence surrounding the ER membrane was altered from the laminar structures to the punctate structures by the GFP-core expression. In contrast to Lro1-mCherry, the GFP-core did not colocalized with Hmg2-mCherry (Fig 8B). Collectively, the core protein might affect the ER membrane and affect the localization of the ER proteins (Hmg2 and Lro1). However, the adjacent localization with a punctate structure of core protein was specific to Lro1, but not to Hmg2 and Dga1.

## Discussion

Yeast is a good model system to study neutral lipid homeostasis [13]. We utilized yeast cells as a model system to study the interaction between an animal virus protein (HCV core) and a eukaryotic cell system [20, 31]. In this study, based on the findings that the core protein had the ability to interact with the ER [20] and LDs (this study) and could induce LD formation in yeast (Fig 1), we performed genetic analyses and identified Lro1 as a factor required for the core-induced LD formation in yeast. We showed that the expression of the core protein might cause changes in the localization of the ER surface protein Lro1 to a position adjacent to LDs (Fig 6B). Despite the Lro1 changes, the distribution of another protein responsible for TAG synthesis, Dga1, was unaffected (Fig 6A). Additionally, the localization of the punctate core was not affected by the loss of Lro1 (Fig 7B). Thus, we speculated that the punctate expression of the core protein on the ER surface might guide the accumulation of Lro1 as patches, which results in LD accumulation (Fig 8C). The mechanism by which Lro1 accumulation occurs remains unknown. We failed to detect the core protein in anti-Myc immunoprecipitates from the lysate of Lro1-Myc expressing yeast (see S6 Fig). This suggested that there might be no direct interaction between Lro1 and the core protein. It should be noted that morphological alterations occurred, and the surrounding punctate LDs were observed in response to core expression in cells in the presence of Lro1. The regulatory mechanism for TAG synthesis via the regulation of Lro1 and Dga1 remains elusive. Nevertheless, our present data suggest that the mobilization of Lro1 may be regulated by a specific mechanism.

Our previous results indicate that upon the induction of LDs, the core is unable to induce the unfolded-protein response [15], suggesting that the core accumulation on the cytoplasmic side of the ER membrane has potential to affect the ER membrane integrity without influencing intra-luminal protein homeostasis. This idea is supported by the fact that the core protein inhibits the degradation of the ER surface protein Lro1, which is likely to be governed by the Hrd1-dependent ERAD system in cells (Fig 5). Thus, an investigation of the retardation of ERAD may be another interesting study on core-dependent events in yeast. Wang and Lee [14] reported that a ubiquitin-like domain containing the Ubx2 protein regulated LD homeostasis by controlling Lro1. Future studies may be interested in determining whether the core protein has an effect on the Ubx2-related system. Collectively, our results suggested that the core accumulation on the cytoplasmic side of the ER membrane changes various aspects of ER homeostasis, namely, the inhibition of ERAD, the alteration of the distribution of ER membrane proteins and the Lro1-dependent accumulation of TAGs (LDs) (see Fig 8C).

Although Lro1 is a homologue of mammalian phosphatidylcholine:cholesterol acyltransferase and lecithin:cholesterol acyltransferase, Lro1 is functionally characterized as a PDAT [32, 33]. DGAT, which uses acyl-CoA as an acyl donor and diacylglycerol as an acceptor to synthesize TAGs, exists in yeast and mammalian cells. In contrast, PDAT, which uses phospholipids as acyl donors, exists in yeasts and plants [13]. Thus, the Lro1-related mechanism identified here may not be fully applicable to the core-dependent LD accumulation in mammalian cells. Previous reports indicated that the core protein appeared to inhibit LD turnover by inhibiting adipose triglyceride lipase (ATGL)-mediated lipolysis in mammalian cells by inhibiting the localization of ATGL and its cofactor [11]. Additional investigations to determine the mechanism responsible for the changes in Lro1 localization by the ER-core interaction in this yeast model system may provide important insights into understanding how the core protein affects the homeostasis of ER surface proteins.

RNA virus genomes evolve quickly because of their high mutation rates to adapt to different circumstances to maintain effective replication. Our observations suggested a functional relationship between the core proteins of viruses with hepatocyte and yeast cells, which in turn implicates an intrinsic interaction of the core D2 region with the ER membrane and possible alterations of the ER membrane.

## Supporting Information

S1 Fig Expression of the core in each of the disruption mutants. (A) and (B): Western blots of cell lysates from the indicated disruption mutant for neutral lipid synthesis. Antibodies against the core and actin were used. (C) and (D) The core levels were normalized using actin (N = 2). The core protein levels relative to actin in A and B, respectively, are shown.
(PDF)

S2 Fig Images of the maximal projection for the observation of BODIPY 493/503-stained LD in yeast mutant cells. (A) The genotypes of yeast strains are indicated in the figure (see the Fig 1 legend). A quadruple disruption mutant (CWY3768), which lacks all four neutral lipid synthesizing genes, is indicated as Δ4. Cells are carrying the empty vector pKT10-GAL (Control) and the pKT10-GAL-core plasmid (Core). LDs in live yeast cells were stained with BODIPY 493/503 and analyzed by fluorescent microscopy. Each image was produced by the maximal projection of ten z-sections at 5 μm thickness. Scale bars: 5 μm. (B) The growth rate of wild-type cells and lro1Δ cells carrying the empty vector pKT10-GAL (-) or the pKT10-GAL-core plasmid (+) upon the condition of induction of core expression. Galactose (3%) was added to exponentially growing yeast cell cultures in SRM (OD_600_ = 0.1). The cultures were further cultured for 6 h. Growth levels after the addition of galactose (6 h) are shown. *P* values are indicated.
(PDF)

S3 Fig Lipid profiles for the core-expressing yeast cells using UHPLC. Lipid species found in wild-type cells, dga1Δ cells and lro1Δ cells carrying the pKT10-GAL-core plasmid before (Raffinose) and after 3 h of culture with galactose (Galactose) by UHPLC analysis are depicted, and their abundances are compared. The fraction of phospholipids includes phosphatidylcholine (PC), phosphatidylethanolamine (PE) and phosphatidylglycerol (PG).
(PDF)

S4 Fig Induction of LDs in cells expressing Lro1-myc with a quadruple mutant. Fluorescent images of BODIPY in Δ4 LRO1-myc cells carrying the empty vector pKT10-GAL (upper panels) or the pKT10-GAL-core plasmid (lower panels, designated as “Core”) after the induction of the core in SRM + Gal medium for 3 h. The DIC images are also shown.
(PDF)

S5 Fig Original results of some blotting. The original Western blotting (A), (B) and (C) for Figs 3, 4 and 5, respectively (see the figure legend).
(PDF)

S6 Fig Immunoprecipitation of Lro1. Wild-type yeast cells carrying the empty (Lro1-myc, -) vector or pRS315-Lro1-myc (Lro1-myc, +) with the empty vector pKT10-GAL (Core, -) or the pKT10-GAL-core (Core, +) were cultured as described above. The cells were collected and resuspended in lysis buffer (50 mM Tris-HCl, pH 7.5, 1 mM EDTA, 150 mM NaCl and 0.1% NP-40) containing 2 mM phenylmethanesulfonyl fluoride, 1 μg/ml leupeptin and 1 μg/ml pepstatin. The cells were then frozen in liquid nitrogen and disrupted by shaking at 2,000 rpm for 30 s with a multi-bead shocker (Yasui Kikai Corporation, Osaka, Japan). The whole cell extract (WCE; 400 μg) was mixed with anti-Myc-Tag agarose (MBL) at 4°C for 3 h. After the beads were extensively washed, the bound proteins were eluted from the beads with 90 μl of sample buffer (50 mM Tris-HCl, pH 6.8, 2% SDS, 0.1% bromophenol blue, and 10% glycerol) with 50 mM DTT. The immunoprecipitates were analyzed by SDS-PAGE (10% and 15% acrylamide gel for Lro1-Myc and Core, respectively) and immunoblotted using anti-Myc rabbit polyclonal antibodies (upper panel) and anti-core mouse monoclonal antibodies (lower panel). The positions of the immunoglobulin heavy chain (HC) and light chain (LC) are indicated.
(PDF)

S1 Table Yeast strain used in this study. (PDF)
