# Supplementary figures and images for: The Phospholipid:Diacylglycerol Acyltransferase Lro1 Is Responsible for Hepatitis C Virus Core-Induced Lipid Droplet Formation in a Yeast Model System
Source: PLoS One. 2016 Jul 26;11(7):e0159324. doi: 10.1371/journal.pone.0159324 (PMC4961368; doi:10.1371/journal.pone.0159324)

S1 Fig

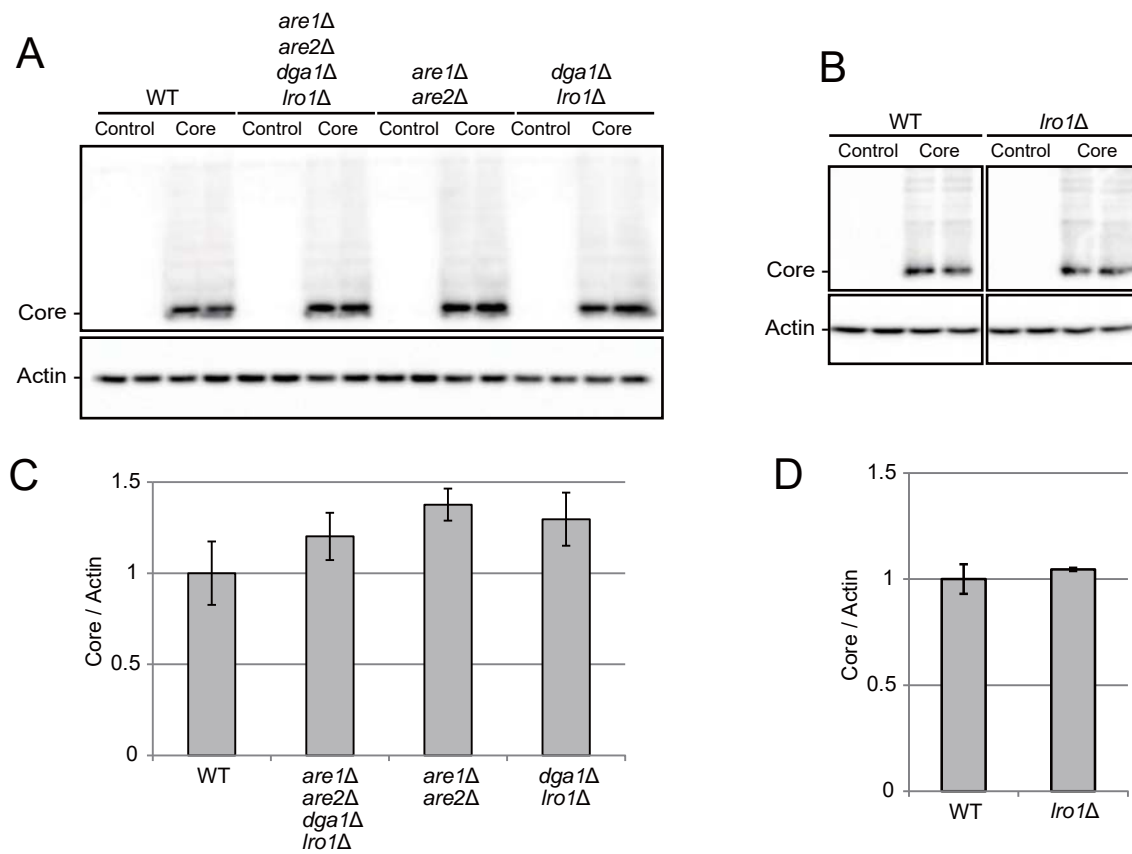

Supplement: S1 Fig — (A) and (B): Western blots of cell lysates from the indicated disruption mutant for neutral lipid synthesis. Antibodies against the core and actin were used. (C) and (D) The core levels were normalized using actin (N = 2). The core protein levels relative to actin in A and B, respectively, are shown. (PDF) [file pone.0159324.s001.pdf]

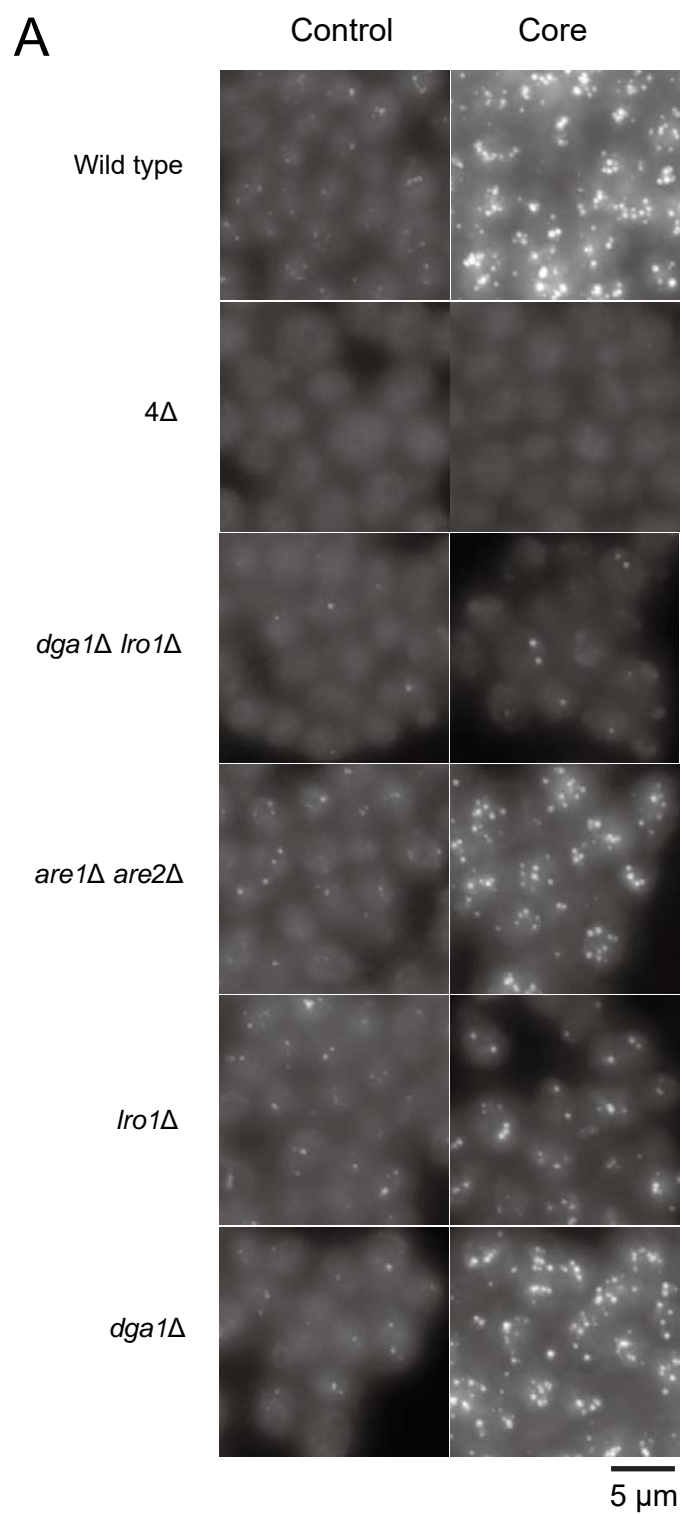

**B** Cell growth 6h after addition of galactose

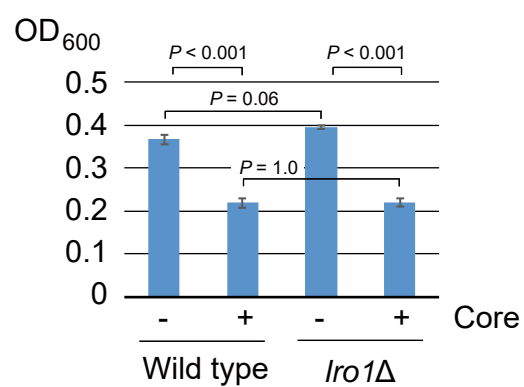

Supplement: S2 Fig — (A) The genotypes of yeast strains are indicated in the figure (see the Fig 1 legend). A quadruple disruption mutant (CWY3768), which lacks all four neutral lipid synthesizing genes, is indicated as Δ4. Cells are carrying the empty vector pKT10-GAL (Control) and the pKT10-GAL-core plasmid (Core). LDs in live yeast cells were stained with BODIPY 493/503 and analyzed by fluorescent microscopy. Each image was produced by the maximal projection of ten z-sections at 5 μm thickness. Scale bars: 5 μm. (B) The growth rate of wild-type cells and lro1Δ cells carrying the empty vector pKT10-GAL (-) or the pKT10-GAL-core plasmid (+) upon the condition of induction of core expression. Galactose (3%) was added to exponentially growing yeast cell cultures in SRM (OD600 = 0.1). The cultures were further cultured for 6 h. Growth levels after the addition of galactose (6 h) are shown. P values are indicated. (PDF) [file pone.0159324.s002.pdf]

S3 Fig

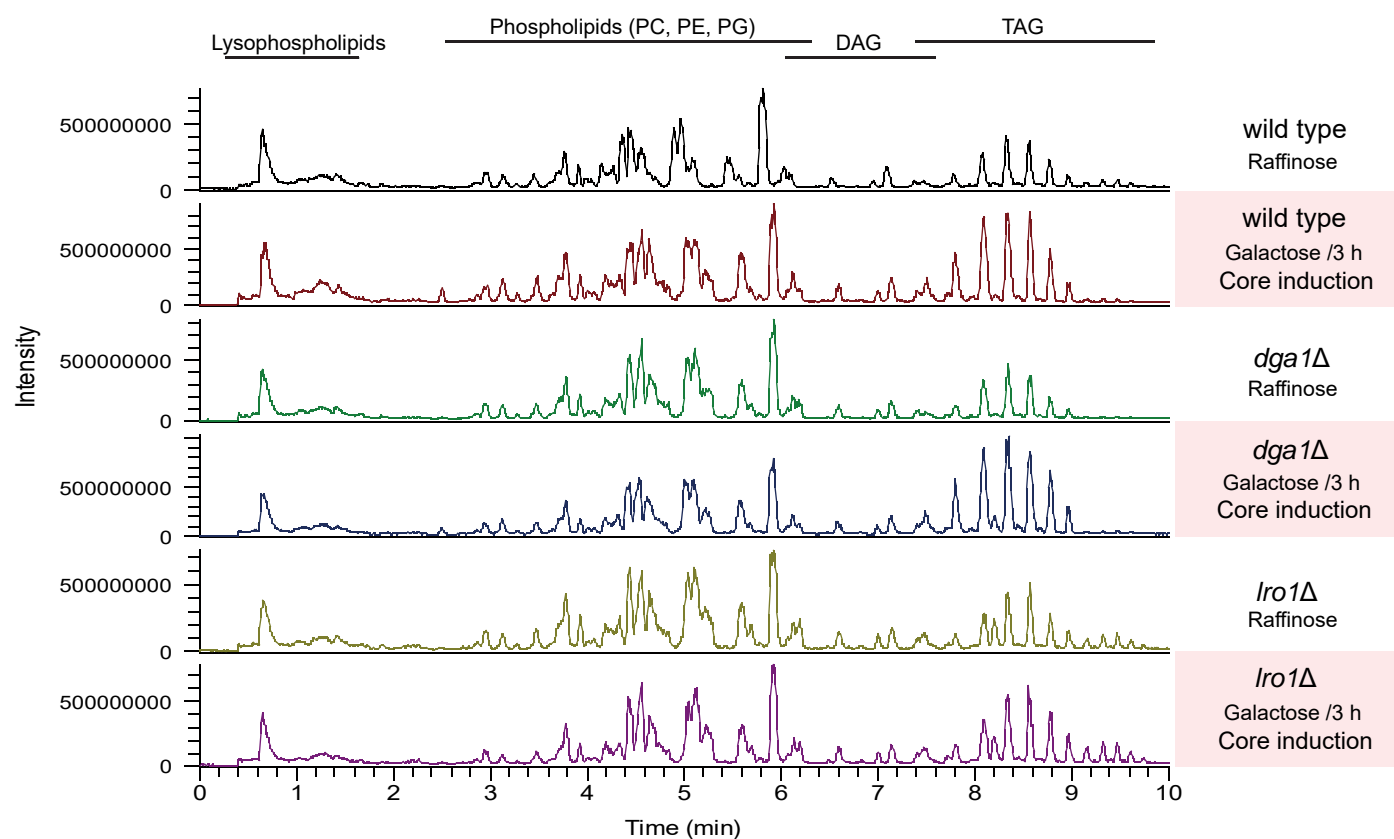

Supplement: S3 Fig — Lipid species found in wild-type cells, dga1Δ cells and lro1Δ cells carrying the pKT10-GAL-core plasmid before (Raffinose) and after 3 h of culture with galactose (Galactose) by UHPLC analysis are depicted, and their abundances are compared. The fraction of phospholipids includes phosphatidylcholine (PC), phosphatidylethanolamine (PE) and phosphatidylglycerol (PG). (PDF) [file pone.0159324.s003.pdf]

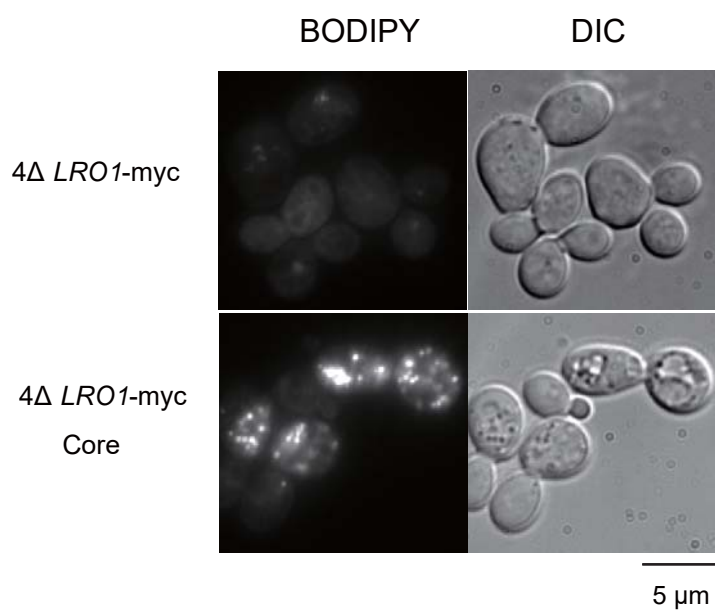

Supplement: S4 Fig — Fluorescent images of BODIPY in Δ4 LRO1-myc cells carrying the empty vector pKT10-GAL (upper panels) or the pKT10-GAL-core plasmid (lower panels, designated as “Core”) after the induction of the core in SRM + Gal medium for 3 h. The DIC images are also shown. (PDF) [file pone.0159324.s004.pdf]

## A CWY3773

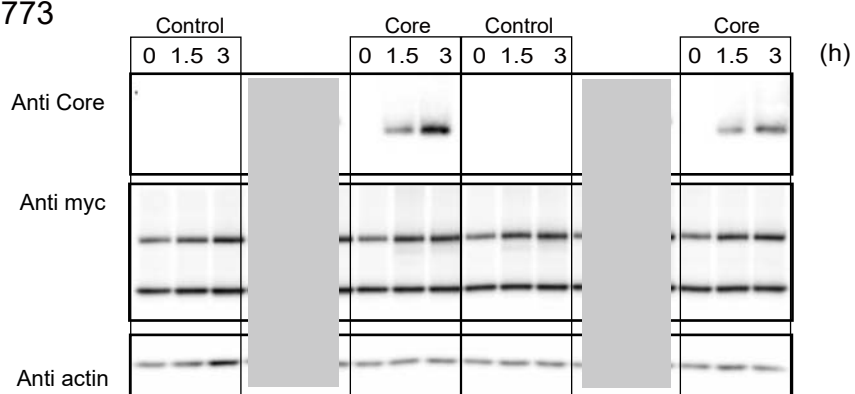

## B CWY3773

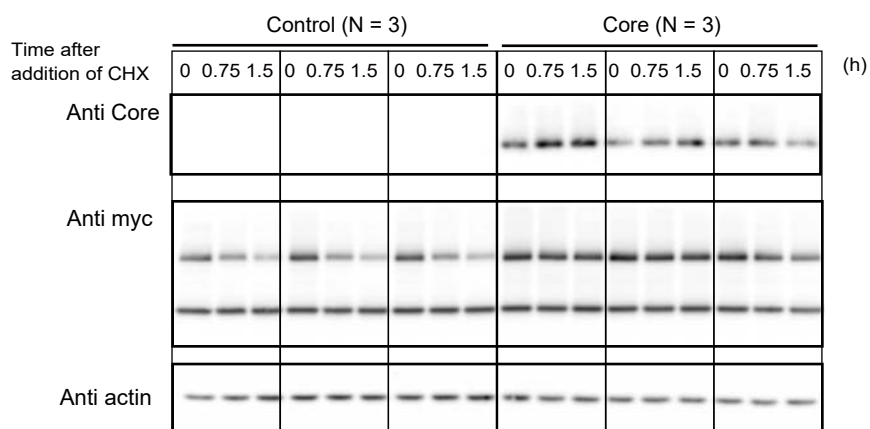

## C Wild type

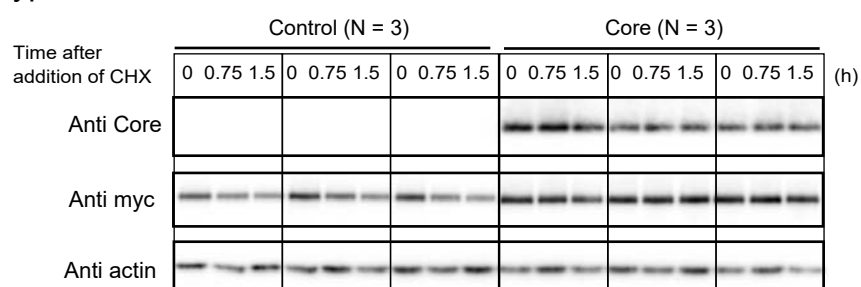*hrd1Δ*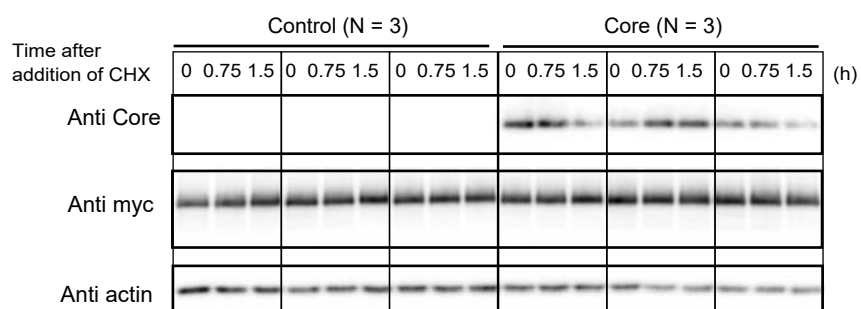

Supplement: S5 Fig — The original Western blotting (A), (B) and (C) for Figs 3, 4 and 5, respectively (see the figure legend). (PDF) [file pone.0159324.s005.pdf]

S6 Fig

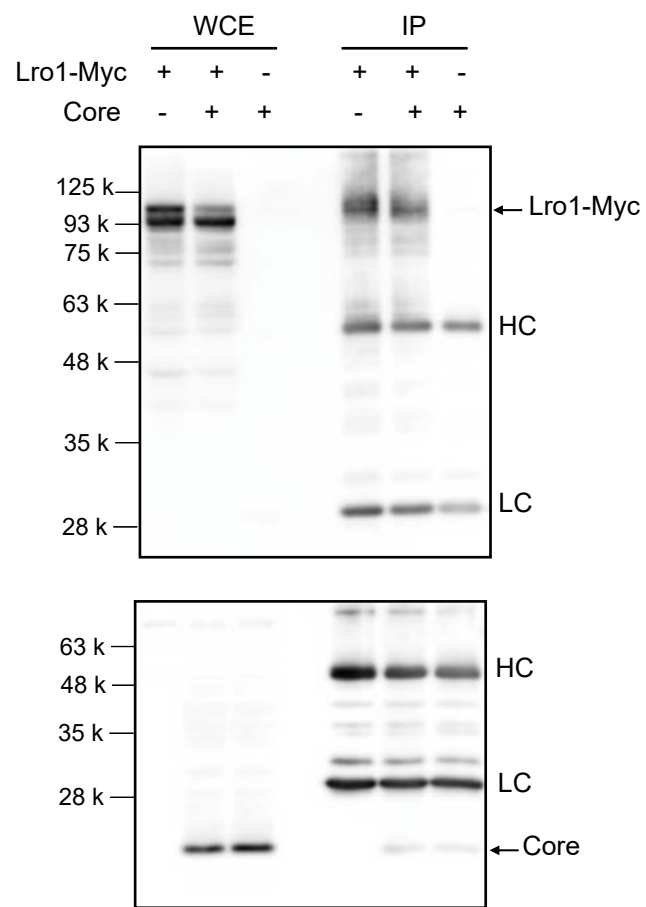

Supplement: S6 Fig — Wild-type yeast cells carrying the empty (Lro1-myc, -) vector or pRS315-Lro1-myc (Lro1-myc, +) with the empty vector pKT10-GAL (Core, -) or the pKT10-GAL-core (Core, +) were cultured as described above. The cells were collected and resuspended in lysis buffer (50 mM Tris-HCl, pH 7.5, 1 mM EDTA, 150 mM NaCl and 0.1% NP-40) containing 2 mM phenylmethanesulfonyl fluoride, 1 μg/ml leupeptin and 1 μg/ml pepstatin. The cells were then frozen in liquid nitrogen and disrupted by shaking at 2,000 rpm for 30 s with a multi-bead shocker (Yasui Kikai Corporation, Osaka, Japan). The whole cell extract (WCE; 400 μg) was mixed with anti-Myc-Tag agarose (MBL) at 4°C for 3 h. After the beads were extensively washed, the bound proteins were eluted from the beads with 90 μl of sample buffer (50 mM Tris-HCl, pH 6.8, 2% SDS, 0.1% bromophenol blue, and 10% glycerol) with 50 mM DTT. The immunoprecipitates were analyzed by SDS-PAGE (10% and 15% acrylamide gel for Lro1-Myc and Core, respectively) and immunoblotted using anti-Myc rabbit polyclonal antibodies (upper panel) and anti-core mouse monoclonal antibodies (lower panel). The positions of the immunoglobulin heavy chain (HC) and light chain (LC) are indicated. (PDF) [file pone.0159324.s006.pdf]
