# Supplementary material for: The Phospholipid:Diacylglycerol Acyltransferase Lro1 Is Responsible for Hepatitis C Virus Core-Induced Lipid Droplet Formation in a Yeast Model System
Source: PLoS One. 2016 Jul 26;11(7):e0159324. doi: 10.1371/journal.pone.0159324 (PMC4961368; doi:10.1371/journal.pone.0159324)
Supplement: S1 Table — (PDF) [file pone.0159324.s007.pdf]

S1 table

Yeast strain used in this study

BY4742	*MATα his3*Δ1 *leu2*Δ0 *lys2*Δ0 *ura3*Δ0	Labo collection
CWY3768	BY4742 *are1Δ::LEU2 are2Δ::HIS3 dga1Δ::KAN lro1Δ::HYG*	Wang and Lee 2012
CWY3773	BY4742 *LRO1-13xMyc::LEU2 DGA1-13xMyc::HIS3*	Wang and Lee 2012
CWY5135	BY4742 *DGA1-mCherry::LEU2 lro1Δ::HYG are1Δ::KAN are2Δ::HIS3*	Wang and Lee 2012
are1/2Δ	BY4742 *are1Δ::LEU2 are2Δ::HIS3*	This study
dga1Δ/lro1Δ	BY4742 *dga1Δ::KAN lro1Δ::HYG*	This study
dga1Δ	BY4742 *dga1Δ::KAN*	This study
lro1Δ	BY4742 *lro1Δ::HYG*	This study
hrd1Δ	BY4742 *hrd1Δ::KAN*	This study
LRO1-mCherry	BY4742 *LRO1-mCherry::LEU2*	This study
HMG2-mCherry	BY4742 *HMG2-mCherry::LEU2*	This study
hrd1Δ LRO1-mCherry	BY4742 *hrd1Δ::KAN* BY4742 *LRO1-mCherry::LEU2*	This study
hrd1Δ HMG2-mCherry	BY4742 *hrd1Δ::KAN HMG2-mCherry::LEU2*	This study
4Δ LRO1-myc	BY4742 *are1Δ::LEU2 are2Δ::HIS3 dga1Δ::KAN LRO1*-myc-Ble	This study
